# The polycomb group protein EZH2 induces epithelial–mesenchymal transition and pluripotent phenotype of gastric cancer cells by binding to PTEN promoter

**DOI:** 10.1186/s13045-017-0547-3

**Published:** 2018-01-15

**Authors:** Lu Gan, Midie Xu, Ruixi Hua, Cong Tan, Jieyun Zhang, Yiwei Gong, Zhenhua Wu, Weiwei Weng, Weiqi Sheng, Weijian Guo

**Affiliations:** 1 Department of Medical Oncology, Fudan University Shanghai Cancer Center, Shanghai, 200032 China; 2 Department of Oncology, Shanghai Medical College, Fudan University, Shanghai, 200032 China; 3 Department of Medical Oncology, Zhongshan Hospital, Fudan University, Shanghai, 200032 China; 4 Department of Pathology and tissue bank, Fudan University Shanghai Cancer Center, Shanghai, 200032 China; 5 Department of Oncology, The First Affiliated Hospital of Sun Yat-Sen University, Guangzhou, Guangdong 510000 China; 6 Department of Pathology, Fudan University Shanghai Cancer Center, Shanghai, 200032 China

**Keywords:** Ezh2, PTEN, Gastric cancer, Pluripotent phenotype, Epithelial–mesenchymal transition

## Abstract

**Background:**

The influences of oncogenic Ezh2 on the progression and prognosis of gastric cancer (GC) and the underlying mechanisms are still poorly understood. Here, we aimed at investigating clinicopathological significance of Ezh2 in GC and the mechanisms underlying its function in GC development.

**Methods:**

The expression level of Ezh2 was determined by qRT-PCR, immunoblot, and immunohistochemistry analysis in 156 pairs of GC tissues and adjacent normal gastric mucosa tissues. The biological functions of Ezh2 were assessed by in vitro and in vivo functional experiments. Chromatin immunoprecipitation (ChIP), luciferase, and Western blotting analyses were utilized to identify the relationship between Ezh2 and the PTEN/Akt signaling.

**Results:**

The expression of Ezh2 was higher in gastric cancer tissues in comparison with para-nontumorous epithelium. High expression of Ezh2 was associated with more aggressive biological behavior and poor prognosis in GC. In vitro studies indicated that Ezh2 promoted GC cells’ proliferation and clonogenicity. Besides, Ezh2 led to the acquisition of epithelial–mesenchymal transition (EMT) phenotype of GC cells and enhanced GC cell migration and invasion capacity. In particular, Ezh2 strengthened sphere-forming capacity of GC cells, indicating its role in the enrichment of GC stem cells. Furthermore, we found that PTEN/Akt signaling contributed to the effects of Ezh2 on cancer stem cells (CSC) and EMT phenotype in GC cells, and blocking PTEN signaling significantly rescued the effects of Ezh2.

**Conclusions:**

Taken together, Ezh2 has a central role in regulating diverse aspects of the pathogenesis of GC in part by involving PTEN/Akt signaling, indicating that it could be an independent prognostic factor and potential therapeutic target.

**Electronic supplementary material:**

The online version of this article (10.1186/s13045-017-0547-3) contains supplementary material, which is available to authorized users.

## Background

Gastric cancer (GC) is one of the highest incident cancers and the leading cause of cancer-related mortality worldwide [1]. In China, GC has become the third highest cause of cancer death, and the relative 5-year survival rate for GC is less than 30% [2, 3]. Adjuvant chemotherapy following a D2 resection is regarded as the standard curative treatments for localized GC, and the standard of care in the first line setting of metastatic disease remains a combination of fluoropyrimidine and platinum-containing chemotherapy [4]. However, the long-term prognosis of GC remains unsatisfactory because of the high rate of recurrence and metastasis. Thus, it is important to investigate the molecular mechanisms of GC progression and to identify genes implicated and the driver mutations/genetic alterations.

The enhancer of zeste homolog 2 (Ezh2) belongs to the family of polycomb group genes (PcGs). It has emerged as a master regulator of cell division [5], DNA repair [6], autophagy [7], signal transduction, and embryonic development [8]. Thus, Ezh2 has multiple essential biological effects, and it is not surprising that its function (and often dysfunction) plays an important role in various diseases, especially in cancer [9]. Increased Ezh2 expression has been reported in different types of cancers, including colorectal cancer [10], hepatocellular carcinomas [11], and lung cancer [12, 13]. Recently Ezh2 protein has been disclosed overexpressed and is associated with several tumor proliferation and invasion-associated genes, as well as the prognosis of GC [14–16]. A panel of genes including HOTAIR [17], CCAT2 [18], and TP53 [19] that implicated in the EMT or pluripotent phenotype have been reported to be acting as the upstream moleculars of Ezh2 dysregulation in GC. These findings raised the question that what the downstream of functional Ezh2 is in cancer EMT phenotype and sphere-forming capacity, resulting in the carcinogenesis and progression of GC.

Herein, we demonstrate that Ezh2 expression is correlated with poor survival in GC. Besides promoting cell proliferation and invasion, Ezh2 has a pivotal role in CSC enrichment and EMT of GC. Most importantly, we show that PTEN is a direct target of Ezh2. Ezh2 binds to the PTEN locus and downregulates PTEN expression, which consequently activates the Akt pathway, stabilizes Vimentin, downregulates E-cadherin, and protects Sox2 and Oct4 from degradation, thus ultimately leads to the acquisition of EMT and pluripotent phenotype of GC cells. Taken together, our results provided an explanation for the aggressive nature of human tumors overexpressing Ezh2 through a mechanism that links Ezh2 to the key tumor suppressor PTEN. These findings of the role of Ezh2 in the progression of GC may imply Ezh2 as a prognostic factor and potential therapeutic target.

## Methods

### Human tissue specimens

The human tissue specimens in this study were collected from 156 patients with histologically confirmed GC, who had underwent prior surgical resection and received no pretreatment. These samples were obtained from the tissue bank of Fudan University Shanghai Cancer Center (FUSCC) between 2008 and 2010. Independently, a total of 105 GC patient samples were enrolled for IHC analysis; all patients’ formalin-fixed paraffin-embedded (FFPE) tissues were obtained from the department of pathology of FUSCC between 2005 and 2011. Follow-up was completed on November 30, 2016. The tumor grades were defined in accordance with the criteria outlined by the World Health Organization (WHO) Classification of Tumors of the Digestive System, 2010 edition [20]. The study complied with the regulations of the Ministry of Health of China and the WHO Research Ethics Review Committee international guidelines for research involving human subjects and the Declaration of Helsinki on the ethical principles for medical research involving human subjects. The survival analysis results of 599 GC tissue samples from GEO cohort (GSE14210, GSE15459, GSE22377, GSE29272, and GSE51105; *n* = 593) are available at the KMPlot database (http://kmplot.com).

### Chromatin immunoprecipitation assays

Chromatin immunoprecipitation (ChIP) assays were performed as previously described [21]. Briefly, the cells were trypsinized and resuspended in lysis buffer, and nuclei were isolated and sonicated to shear the DNA to 500 bp–1 kb fragments (verified by agarose gel electrophoresis). Equal aliquots of chromatin supernatants were subjected to overnight IP with different antibodies as indicated or anti-flag as a negative control. DNA was extracted and the PTEN promoter, as well as the first exon, was amplified by PCR. Sequences of the PCR primers are listed in Additional file 1: Table S6.

### Luciferase assays

Cells were transfected with the pGL3-based constructs containing the PTEN promoter plus the Renilla luciferase plasmid (pRL-TK). Then, the cells were harvested after 48 h for firefly/Renilla luciferase assays using the Dual-Luciferase Reporter Assay System (Promega). Luciferase activities were normalized to the cotransfected pRL-TK plasmid (mean ± SD).

Other methods used in this study were described in previous publications and are listed in the supplementary information [21–24].

### Reproducibility

Each experiment was performed in triplicate, and the data are presented as the mean ± SD. The sphere formation assay, cell invasion assays, animal experiments, Western blot, and immunohistochemistry results are representative of three independent experiments.

### Statistical analysis

All statistical analyses were performed using SPSS 22.0 (IBM, SPSS, Chicago, IL, USA) and GraphPad Prism version 6.0 (GraphPad Software, San Diego, CA, USA). Statistical tests for data between group analysis included the *χ*^2^-test, Student’s two-tailed *t* test, and one-way ANOVA. DFS (disease-free survival) and OS (overall survival) curves were calculated with the Kaplan-Meier method and were analyzed with the log-rank test. The DFS rate was calculated from the date of surgery to the date of progression (local and/or distal tumor recurrence) or to the date of death. The OS rate was defined as the length of time between the diagnosis and death or last follow-up. Univariate and multivariate analysis were fit using a Cox proportional hazards regression model. A threshold of *P* < 0.05 was defined as statistically significant.

## Results

### Ezh2 expression is correlated with the progression and outcome of GC

**Fig. 1:**
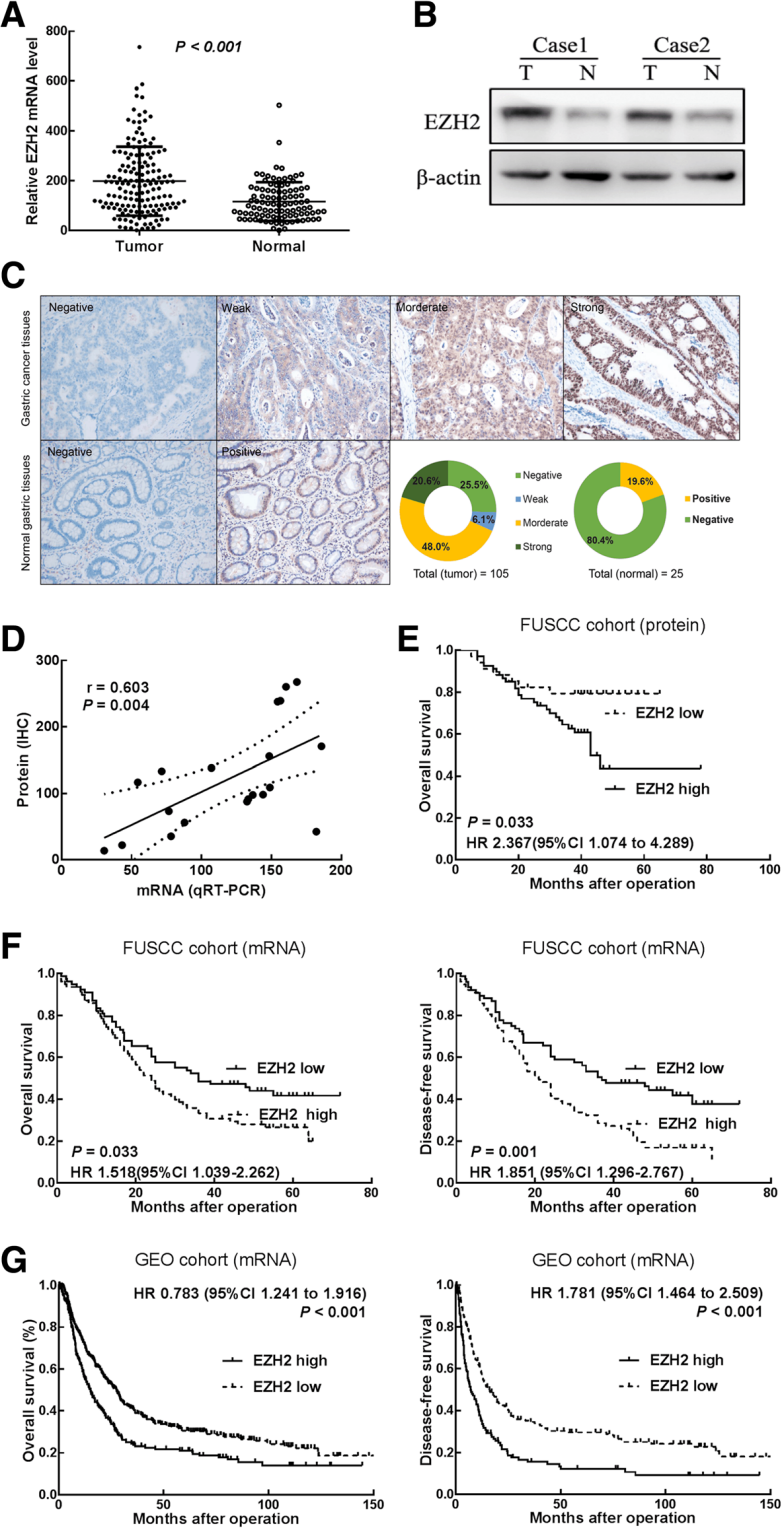
Ezh2 is upregulated in human GC tissues and associated with poor prognosis of GC. **a** The qRT-PCR results showed that the levels of Ezh2 mRNA in 156 pairs of GC tissues were significantly higher than those in adjacent normal mucosa. Data are represented as mean ± SD. **b** Representative band of Western blotting showed that Ezh2 protein expression in GC tissues was higher than those in adjacent normal mucosa. **c** Representative image and the percentage of gastric cancer or normal gastric tissues with low or high Ezh2 protein expression. **d** Correlation analysis showed a positive correlation between EZH2’s mRNA and protein expression in the same 20 cases. Data were analyzed by Spearman’s correlation. **e** Kaplan-Meier survival curves with a log-rank test in the IHC group showed that the OS (*n* = 105) rates of the high EZH2 expression group were significantly lower than that of the low expression group (*P* = 0.033). The HRs and *P* values were calculated with log-rank tests. **f** Kaplan-Meier survival curves showed poor disease-free survival (DFS) and overall survival in patients (FUSCC cohort, *n* = 156) with high expression of Ezh2 compared with those in the low expression group. HRs and *P* values were calculated with log-rank tests. **g** Kaplan-Meier survival curves showed poor disease-free survival (DFS, *n* = 359) and overall survival (*n* = 593) in patients (GEO cohort) with high expression of Ezh2 compared with those in the low expression group. HRs and *P* values were calculated with log-rank tests

To investigate the Ezh2 expression in GC progression, we first examined the expression of Ezh2 by realtime qRT-PCR in 156 cases and immunoblot in randomly selected 20 cases. Ezh2 mRNA and protein expression was higher in GC tissues compared with the matched adjacent normal mucous (Fig. 1a, b). In addition, IHC was employed to examine the protein expression of Ezh2 in 105 cases of primary GC FFPE tissues. Immunoreactivity of Ezh2 was observed primarily in the cytoplasm. The positive expression of Ezh2 protein was observed in 72 (68.6%) cases (Fig. 1c). These findings strongly indicated that Ezh2 is overexpressed in GC. We further analyzed the relationship between qRT-PCR and immunoblot result from the same 20 cases and found a positive correlation between EZH2’s mRNA and protein expression (Fig. 1d).

Then, we analyzed the association between Ezh2 expression and clinicopathological parameters in both qRT-PCR and IHC groups (Additional file 1: Table S1). Ezh2 mRNA expression levels in tumor tissues were categorized as low or high relative based on the median [25]. Statistical analyses revealed that Ezh2 mRNA expression strongly correlated with the tumor size (*P* = 0.003), lymphatic invasion (*P* = 0.019), and TNM stage (*P* = 0.016), indicating that Ezh2 overexpression is associated with the clinical progression of human GC. The results in the IHC cohort showed that Ezh2 protein expression was also correlated with the tumor size (*P* = 0.018), lymphatic invasion (*P* = 0.027) and TNM stage (*P* = 0.038).

To analyze the correlation between Ezh2 mRNA expression and prognosis of GC patients, disease-free survival (DFS) and OS curves were plotted according to Ezh2 expression level by Kaplan-Meier method and the log-rank test. Our result showed that positive Ezh2 protein expression also resulted in a significantly poorer OS in the IHC cohort (*n* = 103, *P* = 0.033, Fig. 1e). Consistently, patients in FUSCC cohort (*n* = 156) with high Ezh2 mRNA expression had a significantly poorer DFS (*P* = 0.001, Fig. 1f) and OS (*P* = 0.033, Fig. 1f) than those with low Ezh2 expression. Moreover, high Ezh2 mRNA expression also resulted in a significantly poorer DFS (*n* = 359, *P* < 0.001; Fig. 1f) and OS (*n* = 593, *P* < 0.001; Fig. 1f) in the GEO cohort (GSE14210, GSE15459, GSE22377, GSE29272, and GSE51105) available at the KMPlot database (http://kmplot.com). Univariate and multivariate Cox proportional hazards analyses showed that Ezh2 and tumor–node–metastasis (TNM) stage were independent prognostic factors for DFS and OS in patients with gastric cancer in the qRT-PCR cohort (Additional file 1: Table S2-3), while only TNM stage was the independent prognostic factor for OS in patients with gastric cancer in the IHC cohort (Additional file 1: Table S4).

### Ezh2 promotes GC cell proliferation and clonogenicity

**Fig. 2:**
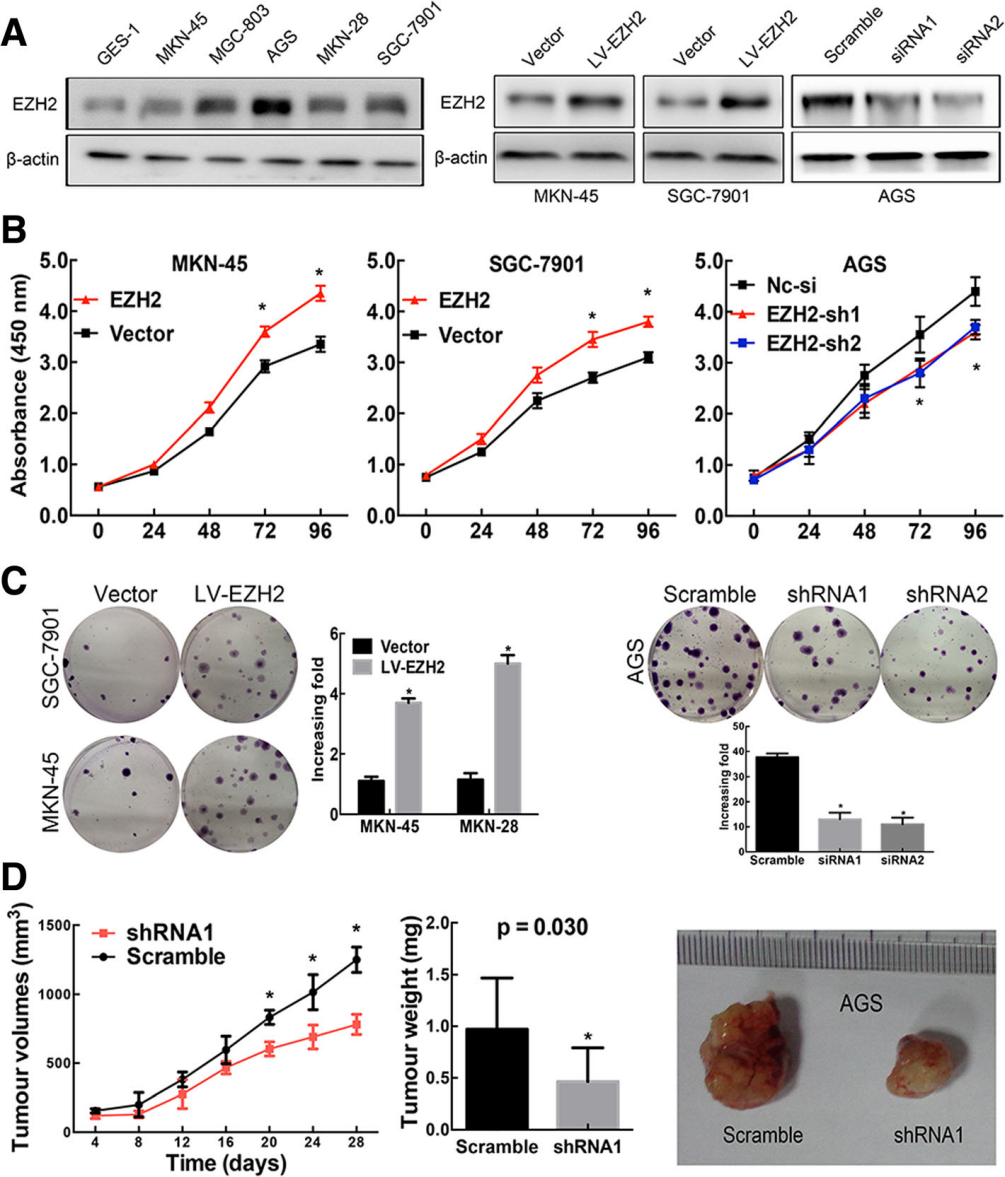
Ezh2 promotes GC cell proliferation, clonogenicity, invasiveness and sphere-forming capacity. **a** The Western blotting results show the baseline protein levels of Ezh2 in GES-1 cell line and five GC cell lines and the efficiencies of Ezh2 overexpression in MKN-45 and SGC-7901 cells and Ezh2 knockdown in AGS cells. *P < 0.01. **b** The CCK-8 assays showed that overexpression of Ezh2 promoted cell proliferation in MKN-45 and SGC-7901 cells, while knockdown of Ezh2 suppressed cell proliferation in AGS cells. Data are represented as mean ± SEM. *P < 0.01. **c** The colony-forming assays showed that overexpression of Ezh2 promoted cell colony-forming in MKN-45 and SGC-7901 cells, while knockdown of Ezh2 suppressed cell colony-forming in AGS cells. Data are represented as mean ± SEM. *P < 0.01. **d** Ezh2*-*knockdown AGS or scrambled-transfected AGS cells were injected into nude mice (*n* = 5) subcutaneously (3 × 10^6^ per mouse). The nude mouse xenograft model showed that knockdown of Ezh2 decreased tumor growth (**d** left) and reduced tumor weights (**d** middle) compared with the scramble groups. Data are represented as mean ± SEM. * *P* < 0.01. The representative images of tumors were graphed (**d** right)

To further investigate the biological role of Ezh2 in GC, we measured the baseline levels of Ezh2 in five GC cell lines and compared with the normal human gastric mucous cell line (GES-1). The Ezh2 level was significantly elevated in GC cells compared with that in the GES-1 cell (all *P* < 0.01, Fig. 2a). Based on the baseline levels of Ezh2, the MKN-45 and SGC-7901 GC cell lines were selected for Ezh2 overexpression, and the AGS cell line was selected for knockdown of Ezh2. The efficiencies of overexpression and knockdown were confirmed by Western blotting (*P* < 0.01, Fig. 2a). We found that Ezh2-overexpressing cells showed a significantly higher in vitro proliferation rate than control cells (*P* < 0.01, Fig. 2b), while knockdown of Ezh2 suppressed cell proliferation in AGS cells in the CCK-8 assays (*P* < 0.01, Fig. 2b) and colony-forming assays (*P* < 0.01, Fig. 2c). Moreover, the mouse xenograft models showed that Ezh2-knockdown cells (AGS-shEzh2) also generated smaller xenografts in nude mice than the control. In accordance with the reported role of Ezh2 in promoting growth, our results indicate that Ezh2 stimulated tumor growth (Fig. 2d).

### Ezh2 induces GC cell sphere-forming capacity and EMT phenotype

**Fig. 3:**
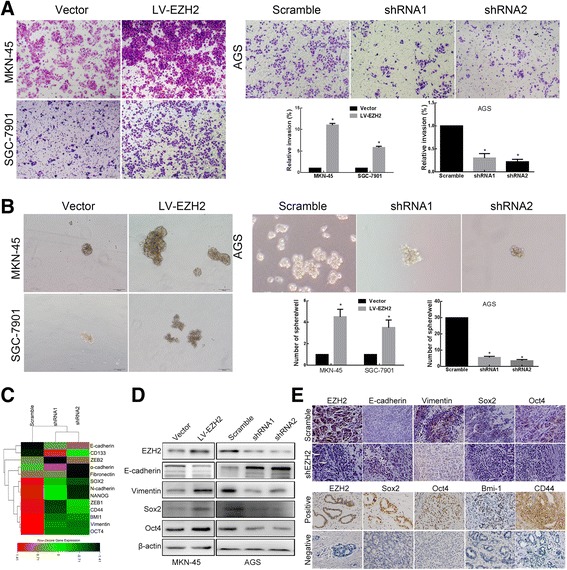
Ezh2 regulates the expression of EMT, pluripotent protein, and PTEN/AKT signaling in GC. **a** Representative images and the number of invasive cells per high-power field (100×) showed that cell invasiveness was promoted by overexpression of Ezh2 in MKN-45 and SGC-7901 cells but suppressed by Ezh2 knockdown in AGS cells. Data are represented as mean ± SEM. *P < 0.01. **b** Representative images and the number of sphere formation per high-power field (100×) showed that stem cell-like properties were promoted by overexpression of Ezh2 in MKN-45 and SGC-7901 cells but suppressed by Ezh2 knockdown in AGS cells. Data are represented as mean ± SEM. *P < 0.01. **c** Heat-map display showed that the Ezh2-altered genes involved in EMT and cell stemness a. Gene expression profiling was performed with mRNA sequencing. **d** Representative images of the Western blot analysis for expression of Ezh2, E-cadherin, Vimentin, Oct4, and Sox2 in Ezh2-overexpressing MKN-45 cells and normal control, as well as Ezh2-knockdown AGS cells and normal control. **e** Representative images of the IHC analysis for expression of Ezh2, E-cadherin, Vimentin, Oct4, and Sox2 in xenograft tissues (up) and expression of Ezh2, Oct4, Sox2, Bmi-1, and CD44 in human GC and normal gastric mucous (down)

Our clinical analysis found that Ezh2 overexpression was significantly correlated with GC tumor lymphatic invasion. We next asked if Ezh2 would affect GC cell invasion. As hypothesized, by the transwell assay, Ezh2-overexpressing cells exhibited a significant increase of invasion, whereas Ezh2 knockdown showed significantly decreased invasion (*P* < 0.01, Fig. 3a).

EMT was a key initial step during tumor invasion. For the detection of the correlation between EZH2- and EMT-related proteins in human gastric cancer, we did mRNA sequencing in EZH2 knockdown cells and then analyzed the gene set enrichment for EMT, which showed a good evidence for EZH2’s regulation on EMT at the molecular level (Fig. 3c). Further validation showed that Ezh2-overexpressing cells exhibited a significant decrease in expression of the epithelial marker E-cadherin and increase in expression of the mesenchymal marker Vimentin, while silencing of Ezh2 expression in AGS cells resulted in increased E-cadherin expression and decreased Vimentin expression (Fig. 3d). These findings demonstrate that Ezh2 could regulate the phenotypic shift of EMT/MET and promote cell invasion. Consistently, immunohistochemical staining demonstrated higher expression of E-cadherin and lower expression of Vimentinin in Ezh2-knockdown tissues of xenograft tumors (Fig. 3e), suggesting that knockdown of Ezh2 attenuates the EMT in vivo.

We also studied the effect of Ezh2 on the stem cell-like phenotype by sphere formation assays. The results showed that Ezh2-overexpressing cells exhibited a significant increase of sphere-forming efficiency, whereas Ezh2 knockdown cells showed significantly decrease sphere-forming efficiency (*P* < 0.01; Fig. 3b). Similarly, mRNA sequencing in EZH2 knockdown cells showed a good evidence for EZH2’s regulation on cell stemness at the molecular level (Fig. 3c), and further validation showed that protein expression levels of the pluripotent genes, Oct4 and Sox2, were both significantly upregulated in Ezh2-overexpressing cells, whereas significantly reduced in Ezh2-knockdown GC cells compared with the control group (*P* < 0.01, Fig. 3d). Consistently, immunohistochemical staining demonstrated higher expression of Oct4 and Sox2 in Ezh2-overexpressing tissues in xenograft tumors (Fig. 3e up). And our IHC results also showed a significant relationship between EZH2 protein and multiple pluripotent phenotype-related proteins (OCT4, BMI1, CD44; all *P* < 0.05) in human GC tissues (Fig. 3e down, Additional file 1: Table S5). All these suggested that Ezh2 enhances the stem cell-like phenotype of GC cells.

### Ezh2 regulates PTEN/AKT signaling by directly binding to the promoter regions of PTEN in GC

It has been reported that the PTEN/AKT signaling is a pivotal signaling pathway involved in EMT; PTEN accumulation in tumor cells might negatively regulate Akt by decreasing Akt phosphorylation level, thus contributes to E-cadherin expression increase and suppression of the EMT [26, 27]. In addition, PTEN and AKT signaling are both implicated in the regulation of cancer stem cell-like phenotype [28, 29]. However, whether Ezh2 promotes tumor cell EMT and enhances the stem cell-like phenotype via AKT/PTEN signaling pathway in gastric cancer warrants validation.

**Fig. 4:**
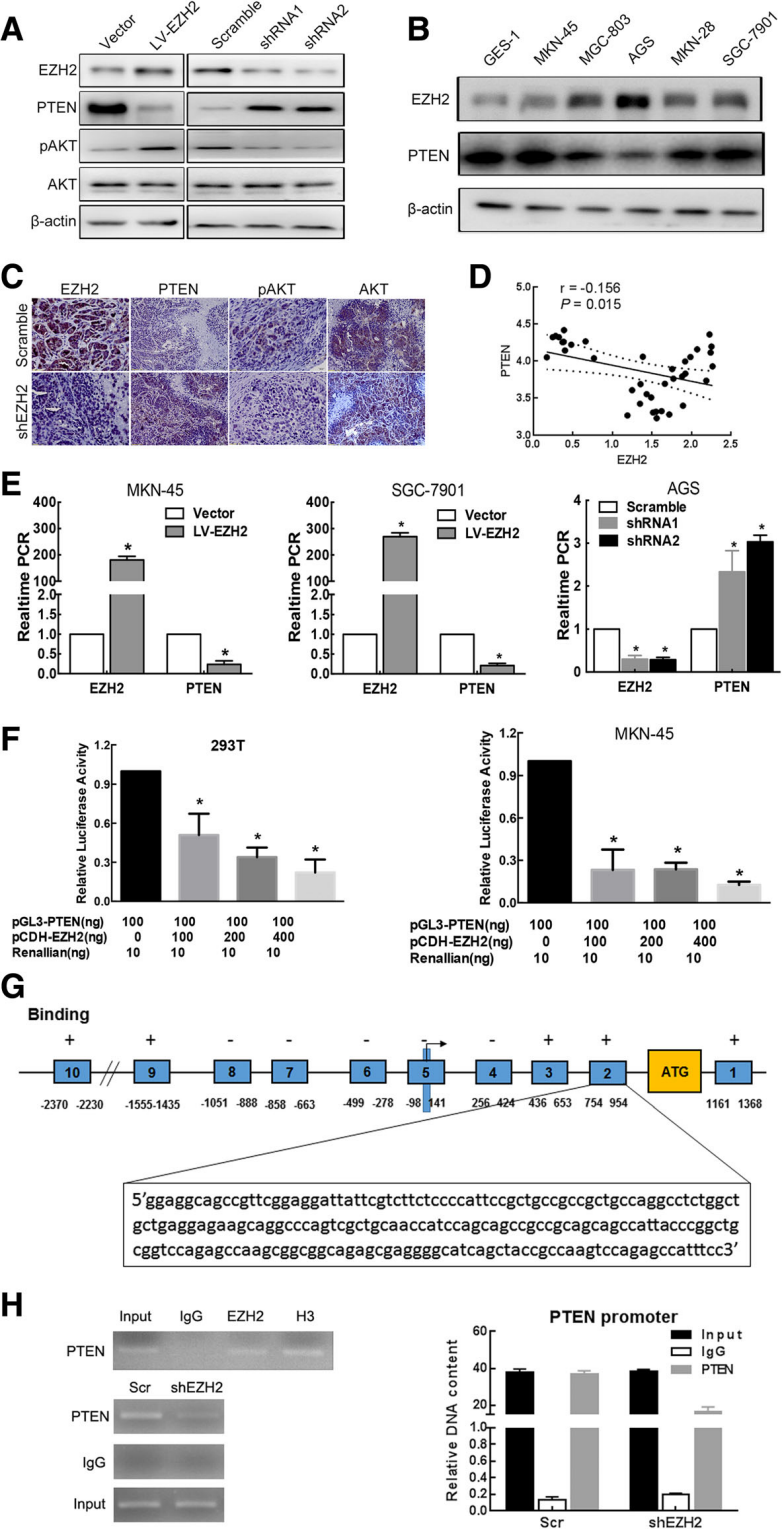
Ezh2 regulates PTEN/AKT signaling by directly binding to the promoter regions of PTEN in GC. **a** Representative images of the Western blot analysis for expression of Ezh2, PTEN, p-Akt, and total Akt in Ezh2-overexpressing MKN-45 and SGC-7901 cells and normal control, as well as Ezh2-knockdown AGS cells and normal control. **b** Representative images of the Western blot analysis for basic expression of Ezh2 and PTEN in five GC cell lines and the normal human gastric mucous cell line (GES-1). **c** Representative images of the IHC analysis for expression of Ezh2, PTEN, p-Akt, and total Akt in xenograft tissues. **d** Ezh2 and PTEN mRNA expression correlation analyses using the *TCGA* gastric cancer data. **e** The qRT-PCR results showed that PTEN mRNA was decreased in Ezh2-overexpressing MKN-45 and SGC-7901 cells, while increased in Ezh2-knockdown AGS cells. Data are represented as mean ± SEM. *P < 0.01. **f** Dual-reporter luciferase assays showed that overexpression of Ezh2 in HEK-293T and MKN-45 cells suppressed the promoter activity of PTEN. Data are represented as mean ± SEM. *P < 0.05. **g** Represent schemata of the PTEN promoter regions with or without binding affinity for EZH2. Arrow indicates the transcriptional start site. ATG indicates translation start codon. **h** ChIP assays showed that endogenous Ezh2 bound to the promoter region of PTEN. IgG served as a negative control, and H3K27 (H3) served as a positive control

In the present study, we found that the Akt phosphorylation and PTEN expression varied with Ezh2 expression, PTEN was significantly decreased while phosphorylated Akt was significantly elevated in Ezh2-overexpressing cells, whereas both expressions were reversed in Ezh2-knockdown cells (Fig. 4a). We then detected the expression of PTEN in different gastric cell lines and find a negative correlation with Ezh2 (Fig. 4b). Consistently, immunohistochemical staining demonstrated lower expression of PTEN and higher level of phosphorylated Akt in Ezh2-knockdown xenograft tumors (Fig. 4c), suggesting that Ezh2 may modulate the AKT/PTEN signaling. Moreover, the expression data in gastric cancer tissue samples from *TCGA* database also reveal a significant negative correlation between Ezh2 and PTEN mRNA in human gastric cancer samples (Fig. 4d).

Considering that Ezh2 is a Polycomb-group (PcG) family member that fulfill its oncogenic functions by participating in maintaining the transcriptional repressive state of genes over successive cell generations, we proposed that Ezh2 may be a transcriptional regulator of PTEN. Then, we determined whether Ezh2 regulates PTEN at the transcriptional level, our qRT-PCR results showed that aberrant expression of Ezh2 indeed affected PTEN mRNA expression; the mRNA levels of PTEN were reduced in Ezh2-overexpressing MKN-45 and MKN-28 cells, while they were increased in Ezh2-knockdown AGS cells (Fig. 4e). We further investigated the regulatory mechanism underlying the correlation between Ezh2 and PTEN by dual-luciferase reporter assays, our results indicated that overexpression of Ezh2 reduced the PTEN promoter activity in HEK-293T and MKN-45 cells (Fig. 4f). ChIP assays were performed to investigate whether Ezh2 associates with the PTEN locus. We proposed that Ezh2 binds to the region of the Pten promoter from − 0.9 to − 2.3 kb and from 0.4 to 1.4 kb; for determination of the exactly binding region, we allocate the Pten promoter into 10 sequences (Fig. 4g, Additional file 1: Table S6). The H3K27 (H3) is antibody serving as positive control, and GAPDH loci were used as internal ChIP control for Ezh2 unbound region. The results revealed that Ezh2 directly binds to the PTEN promoter from 754 to 954 bp (Fig. 4h), suggesting that Ezh2 directly binds to the PTEN promoter to regulate its transcription.

### PTEN/Akt acts as the downstream component of Ezh2 and contributes to the effects of Ezh2 on the pluripotent and EMT phenotype in GC cells

**Fig. 5:**
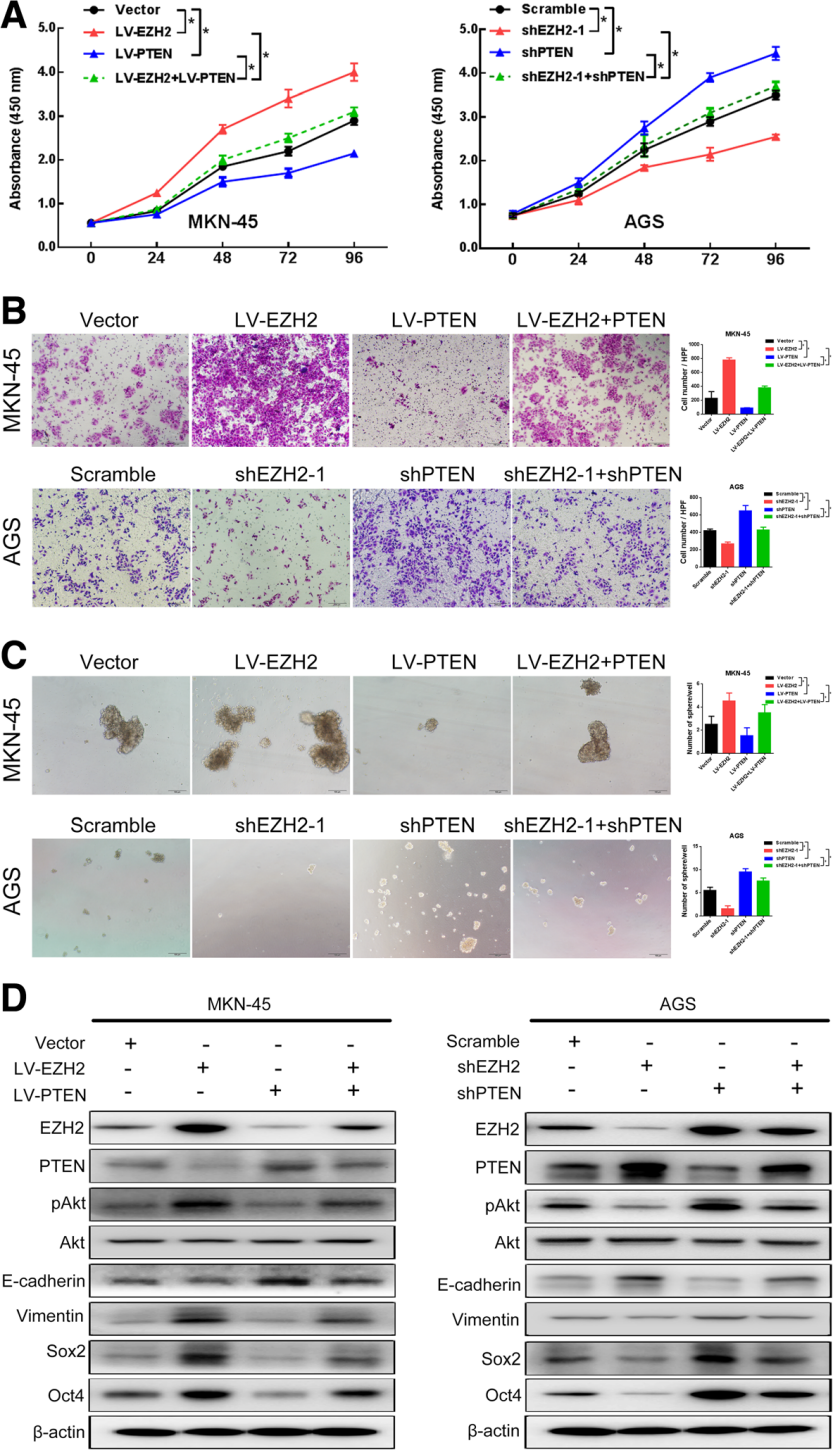
Ezh2 facilitated GC progression in part via PTEN/Akt pathway. **a** The CCK-8 results showed that overexpression of PTEN partially attenuated the enhanced cell proliferation induced by overexpression of Ezh2 in MKN-45 cells, while knockdown of PTEN partially rescued the decreased cell proliferation induced by knockdown of Ezh2 in MKN-45 cells. Data are represented as mean ± SEM. * *P* < 0.01. **b** Representative images (left) and the number of invading cells (right) per high-power field showed that upregulation of PTEN partially attenuated the enhanced invasiveness ability of MKN-45 cells promoted by Ezh2 overexpression, while downregulation of PTEN partially rescued the decreased cell invasiveness of AGC cells promoted by Ezh2 knockdown. Data are represented as mean ± SEM. * *P* < 0.01. **c** Representative images (left) and the number of sphere formation (right) per high-power field showed that upregulation of PTEN partially attenuated the enhanced sphere formation of MKN-45 cells promoted by Ezh2 overexpression, while downregulation of PTEN partially rescued the decreased sphere formation activity of AGS cells promoted by Ezh2 knockdown. Data are represented as mean ± SEM. * *P* < 0.01. **d** Ezh2-overexpressing MKN-45 cells were transfected with empty vector or LV-PTEN, while Ezh2-knockdown AGS cells were transfected with scrambled or PTEN siRNA, and cells were subjected to western blotting with the indicated antibodies

Finally, we conducted in vitro experiments to investigate whether Ezh2 functioned in a PTEN-mediated manner in GC. The enhanced or suppressed proliferative, invasion, sphere formation capacity in GC cells caused by overexpressing or knockdown Ezh2 was significantly attenuated or restored by upregulation or downregulation of PTEN (Fig. 5a–c). And protein expression levels of the EMT and pluripotent genes (E-cadherin, Vimentin, and Sox2 were both in consistent with the cellular biological behavior (Fig. 5d).

We further tested the change of AKT signaling and found that Ezh2 overexpression can induce AKT phosphorylation, while simultaneous overexpression of PTEN can suppress AKT phosphorylation (Fig. 5d). Similarly, when Ezh2 is silenced, pAKT is downregulated, while knockdown of PTEN at the same time can restore the expression of pAKT (Fig. 5d). These data indicated that upregulation of Ezh2 activates the AKT/PTEN pathway in GC cells. Our results indicated that Ezh2 induce the EMT and pluripotent phenotype of GC cell in a AKT/PTEN-mediated manner.

## Discussion

In this study, we determined the pivotal role of Ezh2 in GC pathogenesis and its underlying mechanisms. We observed that elevated Ezh2 expression was directly correlated with GC tumorigenesis and progression. High expression of Ezh2 could be used to identify a greatly increased risk of recurrence or invasion in patients after surgery, which might serve as a valuable prognostic marker. We also found that in addition to facilitating the proliferation and invasion of GC cells, Ezh2 especially lead to the EMT and pluripotent phenotype of GC cells through mediating the Akt/PTEN signaling pathway. Collectively, our clinical and mechanistic evidence strongly suggested that dysregulated Ezh2 expression mediating abnormal Akt/PTEN signaling critically contributes to GC pathogenesis and aggressive behavior.

The present study demonstrated that Ezh2 was overexpressed in GC specimens. Similar results have been obtained by other investigations, although the number of tumors analyzed was different and those studies focused on only the protein level [30, 31]. These findings provide support for a role of elevated Ezh2 protein expression in the tumorigenesis of GC. More importantly, we demonstrate for the first time the potential role of deregulated Ezh2 mRNA in promoting GC progression and unfavorable prognosis. We demonstrated that Ezh2 mRNA expression is significantly correlated with tumor stage and invasion. Further analysis demonstrated that Ezh2 mRNA, while not EZH2 protein expression, was an independent prognostic indicator for GC survival, strongly suggesting that GC patients with high Ezh2 expression should undergo follow-up at shorter intervals and be kept under close surveillance.

Our studies demonstrated that the toggle points protein of Akt signaling, PTEN, is transcriptionally regulated by Ezh2 in GC partly and contributes to the high-proliferation and invasiveness activity, EMT, and pluripotent phenotype. It was previously identified that PTEN/Akt signaling [32] and Ezh2 [33] are important regulators for the proliferation and invasion of gastric cancer cells. Here, we present a mechanism in consistent with these previous observations. Previous studies established the critical roles of PTEN/Akt signaling activity in tumor cell pluripotent phenotype [34]. The present study is the first to demonstrate that PTEN/Akt signaling activity is associated with Ezh2 activation in human GC, suggesting a link between PTEN/Akt signaling inactivation and Ezh2 activation. By using well-established Ezh2 upregulation and downregulation systems, we were able to show that aberrant expression of Ezh2 significantly impacted PTEN expression and Akt phosphorylation in GC cells. We also found that Ezh2 expression is involved in PTEN promoter activity in GC cells, and the binding is functional, which we confirmed by using luciferase and ChIP assay and rescue experiments. However, Ezh2 is a histone methyltransferase and the catalytic subunit of the Polycomb Repressive Complex 2 (PRC2), but not a transcription factor that binds a specific DNA sequence/motif. Thus, there should be other factors that help Ezh2 to bind to PTEN promoter. It has been found that imatinib could cause drug resistance via recruitment of EZH2 to the promoter region of the PTEN and then downregulates PTEN’s transcripts in leukemia patients [35]; moreover, cumulative evidences revealed that lncRNAs interacted with EZH2 and recruited it to genes’ promoter regions to repress genes’ expression [36–38]. Furthermore, Chen NM and colleagues found that KRAS signaling was required for EZH2-mediated transcriptional activation of the inflammatory transcription factor nuclear factor of activated T cells 1 (NFATC1) in pancreatic ductal adenocarcinoma cells [39]. All these suggest that drugs, lncRNA, and transcription factor, may contribute to the binding of EZH2 to PTEN promoter. Further studies may focus on the exactly molecular contribution to EZH2 recruitment to the promoter region of PTEN.

Besides its aforementioned role, the implication of a role for Ezh2 in GC tumorigenesis and development is supported by lines of evidence suggesting that Ezh2 is essential for cellular EMT in GC [40–42]. In fact, Ezh2 regulates the expression of various EMT-related genes in various cancer types [40–42], suggesting that Ezh2 is required for EMT during tumor progression. In addition to the present study is the first to provide evidence of a critical role for activated Ezh2 in gastric cancer pluripotent phenotype by regulating PTEN/Akt signaling; it also shows a tight correlation between Ezh2 expression and E-cadherin and Vimentin expression mediate by PTEN/Akt signaling, supporting a critical role for Ezh2-PTEN/Akt axis in cellular EMT. Given the identified pivotal roles of Ezh2 in these two critical aspects of cancer biology, promotion of GC progression by activation of Ezh2 is conceivable.

Aberrant Ezh2 expression occurs in many other solid tumors, but its effects and underlying mechanism in these cancers are currently unclear. Whether Ezh2 is involved in the oncogenesis and development of other human malignancies via the PTEN/Akt signaling is still worth exploring, and the results would help us to better understand the role of Ezh2 in cancer.

## Conclusion

In summary, we demonstrated that Ezh2 is overexpressed in primary GC and its expression is correlated with the tumor burden and clinical outcome. In addition, besides promoting GC cell proliferation and invasion, we identified Ezh2 as a key regulator of GC cell EMT and pluripotent phenotype via activation of the PTEN/Akt pathway. Our data strongly suggested that Ezh2 can be used for prognostication for GC and it may be utilized as a therapeutic target. Further exploration is necessary for the mechanism underlying Ezh2 overexpression in GC, particularly the molecule(s) to which it binds when modulating tumor cell behavior. Therefore, Ezh2 provides new perspectives for research of cell regulation in gastric cancer and new targets for gastric cancer diagnosis and treatment.

## Additional file

Additional file 1: Materials and Methods. **Table S1.** Relationship between EZH2 expression and clinicopathologic parameters of gastric cancer patients. **Table S2.** Univariate and multivariate analysis of clinicopathological factors for disease-free survival in gastric cancer (qRT-PCR cohort). **Table S3.** Univariate and multivariate analysis of clinicopathological factors for overall survival in gastric cancer (qRT-PCR cohort). **Table S4.** Univariate and multivariate analysis of clinicopathological factors for overall survival in gastric cancer (IHC cohort). **Table S5.** Correlation analysis of expression of stem cell related factors with EZH2. **Table S6.** Primers and siRNA sequences used in this study. (DOCX 67 kb)
