# Supplementary material for: The polycomb group protein EZH2 induces epithelial–mesenchymal transition and pluripotent phenotype of gastric cancer cells by binding to PTEN promoter
Source: J Hematol Oncol. 2018 Jan 15;11:9. doi: 10.1186/s13045-017-0547-3 (PMC5769437; doi:10.1186/s13045-017-0547-3)
Supplement: Additional file 1: — Materials and Methods. Table S1. Relationship between EZH2 expression and clinicopathologic parameters of gastric cancer patients. Table S2. Univariate and multivariate analysis of clinicopathological factors for disease-free survival in gastric cancer (qRT-PCR cohort). Table S3. Univariate and multivariate analysis of clinicopathological factors for overall survival in gastric cancer (qRT-PCR cohort). Table S4. Univariate and multivariate analysis of clinicopathological factors for overall survival in gastric cancer (IHC cohort). Table S5. Correlation analysis of expression of stem cell related factors with EZH2. Table S6. Primers and siRNA sequences used in this study. (DOCX 67 kb) [file 13045_2017_547_MOESM1_ESM.docx]

**Additional file 1 Material and methods**

**Reagents and antibodies**

Luciferase reporter gene constructs containing the PTEN promoter were cloned into the promoter-less pGL3 enhancer plasmid vector. The following antibodies were used: mouse anti-EZH2 (Abcam), mouse anti-E-cadherin and Vimentin (Transduction Laboratories), mouse anti-PTEN and anti-β-actin (Santa Cruz Biotechnology Inc), rabbit-anti-Akt or p-Akt (Cell Signaling). Anti-flag (Sigma) secondary antibodies were conjugated to FITC or rhodamine (goat anti-rabbit IgG or goat anti-mouse IgG) (Jackson Laboratory) or Peroxidase (goat anti-rabbit IgG or goat anti-mouse IgG) (Amersham Pharmacia Biotech). The primers and DNA oligos used in this study are listed in Supplemental Table S5.

**Cell line and culture medium**

Human gastric cancer cell lines (AGS, MGC-803, MKN-28, MKN-45 and SGC7901) and a normal human gastric mucous cell line (GES-1) were cultured was performed as previously described [[22](#_ENREF_22)]. Stable MKN-45 and SGC7901 cells transfected with pLV-ZsGreen-puro (pLV) constructs were grown in 2 μg/ml puromycin (Cat. No. 631306, Clontech, USA), and the stable Ezh2 transfected MKN-45 and SGC7901 cell lines transfected with pLVX-PTEN-G418 constructs were maintained with 400 μg/ml G418 (Cat. No. 631308, Clontech, USA).

**Western blot analysis, RNA extraction and qRT-PCR**

Western blot analysis and qRT-PCR was performed as previously described [[21](#_ENREF_21)].

**Overexpression and knockdown**

The pLV- Ezh2, pLVX-PTEN and Ezh2/pten short hairpin RNA (shRNA) constructs were generated as described previously [[21](#_ENREF_21)]. Cell transfection was performed as previously described [[42](#_ENREF_42)]. All transfected cells were selected with puromycin (2 mg/ml) or G418 (600 μg/ml) for two weeks. The stable transfected cell lines were identified using qRT-PCR.

**Cell proliferation assay, colony formation assay and cell invasion assays**

Cell proliferation assay and colony formation assay were performed as previously described [2[4](#_ENREF_42)].

**Tumor Model**

All procedures involving animal experiments were approved by the Shanghai Medical Experimental Animal Care Commission. Male nude mice were maintained under specific pathogen-free conditions in the Experimental Animal Department of Fudan University. All of the experimental procedures involving animals were undertaken in accordance with the institute guidelines. For the tumorigenicity studies, AGS cells expressing control shRNA or shEzh2 (3×10^6^) were subcutaneously injected into the dorsal flank of 4-week-old mice (Shanghai SLAC Laboratory Animal Co. Ltd.) (n = 5 mice per group). Tumors were examined twice weekly; the length and width were measured using calipers, and the tumor volumes were calculated using the following equation: (L*W^2^)/2. When the mice were sacrificed, the tumors were dissected, weighed and paraffin embedded. Sections of the tumors were cut and subjected to immunohistochemical staining as previously described [[23](#_ENREF_23)].

**Tissue microarray (TMA) immunohistochemistry assay**

The 10×12 TMA was made by FUSCC Tissue Bank. IHC was performed on 5-μm-thick TMA sections using the antibody against EZH2. Each case has two cores made from separate sources to preclude the heterogeneity or loss of tumors. A known positive case sample was included as a positive control, and the primary antibody was replaced with non-immune mouse/rabbit serum for negative control. The immunoreactive Score (IRS) was multiplicity of the staining intensity and positive cancer percentage. Finally, the assessment of the protein expression was defined as low (≤1+) and high (>1+).

**Table S1** Relationship between EZH2 expression and clinicopathologic parameters of gastric cancer patients

| Clinicopathologic feature | qRT-PCR group | | P value^b^ | IHC group | | P value^b^ |
| --- | --- | --- | --- | --- | --- | --- |
|  | N | % |  | N | % |  |
| Age(years) |  |  | 0.559 |  |  | 0.307 |
| <60 | 70 | 44.9 |  | 38 | 36.2 |  |
| ≥60 | 86 | 55.1 |  | 57 | 63.8 |  |
| Gender |  |  | 0.231 |  |  | 0.983 |
| male | 120 | 76.9 |  | 67 | 65.0 |  |
| female | 36 | 23.1 |  | 28 | 35.0 |  |
| Tumor mass size |  |  | 0.003 |  |  | 0.018 |
| <5cm | 103 | 66.0 |  | 66 | 62.9 |  |
| ≥5cm | 53 | 34.0 |  | 39 | 37.1 |  |
| Histologic grade |  |  | 0.096 |  |  | 0.977 |
| Well and moderately | 118 | 75.6 |  | 52 | 49.5 |  |
| Poorly and others | 38 | 24.4 |  | 53 | 50.5 |  |
| Depth of tumor |  |  | 0.267 |  |  | 0.926 |
| T1 and T2 | 11 | 7 |  | 25 | 23.8 |  |
| T3 and T4 | 145 | 93 |  | 80 | 76.2 |  |
| Vascular invasion |  |  | 0.065 |  |  | 0.056 |
| Absent | 54 | 34.6 |  | 60 | 57.1 |  |
| Present | 102 | 65.4 |  | 45 | 42.9 |  |
| Nervous invasion |  |  | 0.305 |  |  | 0.051 |
| Absent | 52 | 33.3 |  | 57 | 54.3 |  |
| Present | 104 | 66.7 |  | 38 | 45.7 |  |
| Lymphatic metastasis |  |  | 0.019 |  |  | 0.027 |
| Absent | 17 | 10.9 |  | 31 | 29.5 |  |
| Present | 139 | 89.1 |  | 74 | 70.5 |  |
| TNM stagea |  |  | 0.016 |  |  | 0.038 |
| I and II | 27 | 17.3 |  | 26 | 24.7 |  |
| III and IV | 129 | 82.7 |  | 79 | 75.3 |  |

^a^ Tumor stage was obtained according to the TNM criteria.

^b^ All statistical tests were 2-sided. Significance level: P < 0.05.

**Table S2** Univariate and multivariate analysis of clinicopathological factors for disease-free survival in gastric cancer (qRT-PCR cohort)

| Variable | Univariate analysis | | | Multivariate analysis | | |
| --- | --- | --- | --- | --- | --- | --- |
|  | HR (95 % CI) | pa | HR (95 % CI) | | pa |  |
| Age  (<60/≥60) | 0.956 (0.667-1.371) | 0.808 |  | |  |  |
| Gender  (Male/Female) | 0.982 (0.638-1.513) | 0.935 |  | |  |  |
| Tumor size  (<5/≥5) | 1.022 (0.689-1.518) | 0.913 |  | |  |  |
| Histologic grade  (well,mod/poor,others) | 0.708 (0.450-1.114) | 0.136 |  | |  |  |
| Depth of tumor  (T1,T2/T3,T4) | 2.857 (1.051-7.762) | 0.040 |  | |  |  |
| Vascular invasion  (Absent/Present) | 1.354 (0.904-2.029) | 0.142 |  | |  |  |
| Nervous invasion  (Absent/Present) | 1.399 (0.930-2.104) | 0.107 |  | |  |  |
| Lymphatic metastasis  (Absent/Present) | 2.503 (0.998-4.222) | 0.051 |  | |  |  |
| TNM stage  (I+II/III+IV) | 2.125 (1.189-3.789) | 0.011 | 1.916 (1.065-3.446) | | 0.030 |  |
| EZH2 mRNA  (low/high) | 1.795 (1.227-2.627) | 0.002 | 1.670 (1.137-2.452) | | 0.009 |  |

HR Hazard ratio, CI confidence interval,

^a^ All statistical tests were 2-sided. Significance level: P < 0.05.

**Table S3** Univariate and multivariate analysis of clinicopathological factors for overall survival in gastric cancer (qRT-PCR cohort)

| Variable | Univariate analysis | | Multivariate analysis | |
| --- | --- | --- | --- | --- |
|  | HR(95 % CI) | pa | HR(95 % CI) | pa |
| Age  (<60/≥60) | 1.090 (0.75-1.584) | 0.651 |  |  |
| Gender  (Male/Female) | 1.061 (0.680-1.657) | 0.793 |  |  |
| Tumor size  (<5/≥5) | 1.096 (0.730-1.646) | 0.658 |  |  |
| Histologic grade  (well,mod/poor,others) | 0.678 (0.419-1.096) | 0.113 |  |  |
| Depth of tumor  (T1,T2/T3,T4) | 3.270 (1.036-10.324) | 0.043 |  |  |
| Vascular invasion  (Absent/Present) | 1.411 (0.921-2.162) | 0.113 |  |  |
| Nervous invasion  (Absent/Present) | 1.442 (0.937-2.219) | 0.096 |  |  |
| Lymphatic metastasis  (Absent/Present) | 2.470 (1.081-5.643) | 0.032 |  |  |
| TNM stage  (I+II/III+IV) | 2.561 (1.331-4.930) | 0.005 | 2.405 (1.243-4.562) | 0.009 |
| EZH2 mRNA  (low/high) | 1.521 (1.027-2.252) | 0.033 | 1.396 (1.183-2.074) | 0.048 |

HR Hazard ratio, CI confidence interval,

^a^ All

**Table S4** Univariate and multivariate analysis of clinicopathological factors for overall survival in gastric cancer (IHC cohort)

| Variable | Univariate analysis | | | Multivariate analysis | | |
| --- | --- | --- | --- | --- | --- | --- |
|  | HR(95 % CI) | p^a^ | HR(95 % CI) | | p^a^ |  |
| Age  (<60/≥60) | 1.196 (0.598-2.391) | 0.808 |  | |  |  |
| Gender  (Male/Female) | 0.884 (0.426-2.085) | 0.935 |  | |  |  |
| Histologic grade  (well,mod/poor,others) | 0.651 (0.294-1.441) | 0.136 |  | |  |  |
| Depth of tumor  (T1,T2/T3,T4) | 1.776 (0.542-5.817) | 1.776 |  | |  |  |
| Vascular invasion  (Absent/Present) | 1.704 (0.828-3.507) | 0.148 |  | |  |  |
| Nervous invasion  (Absent/Present) | 1.193 (0.601-2.367) | 0.613 |  | |  |  |
| Lymphatic metastasis  (N1+2/ > N3) | 2.291 (1.158-4.533) | 0.017 |  | |  |  |
| TNM stage  (I+II/III+IV) | 2.995 (1.180-7.602) | 0.011 | 2.995 (1.180-7.602) | | 0.021 |  |
| EZH2 protein  (low/high) | 2.404 (1.040-5.556) | 0.040 |  | |  |  |

HR Hazard ratio, CI confidence interval,

^a^ All statistical tests were 2-sided. Significance level: P < 0.05.

**Table S5** Correlation analysis of expression of stem cell related factors with EZH2

|  | Sox2 | Oct4 | Bmi-1 | CD44 |
| --- | --- | --- | --- | --- |
| R | 0.117 | 0.168 | 0.217 | 0.244 |
| P | 0.125 | 0.027 | 0.004 | 0.001 |
| R: Spearman correlation coefficient, Significance level: P ＜0.05. | | | | |

**Table S6** Primers and siRNA sequences used in this study

Primers for qRT-PCR

| Primer name 5′-3 | 5′-3′ |
| --- | --- |
| EZH2-F | AGCCGCTGACCATTGGGACAGTA |
| EZH2-R | CTCTCCACAGTATTCTGAGATG |
| PTEN-F | GGAGTAACTATTCCCAGTCAGAGG |
| PTEN-R | CTTCACCTTTAGCTGGCAGACCAC |
| β-actin-F | ATCATGTTTGAGACCTTCAA |
| β-actin-R | CATCTCTTGCTCGAAGTCCA |

Primers for ChIP assay

| Primer name 5′-3 | 5′-3′ |
| --- | --- |
| PTEN -left (754~954) | 5' ggaggcagccgttcggaggattatt3' |
| PTEN -right | 5' ggaaatggctctggacttggcggta 3' |
| GAPDH-left | 5' ggtagggagttcgagaccag 3' |
| GAPDH-right | 5' tcaacgcagttcagttaggc 3' |

Primers for subcloning and plasmid construction

| Primer name 5′-3 | 5′-3′ |
| --- | --- |
| pCDNA3.1/EZH2-F | ataggatccATGGGCCAGACTGGGAAGAAATCT |
| pCDNA3.1/EZH2-R | atactcgagTCAAGGGATTTCCATTTCTCTTTC |
| pCDNA3.1/PTEN-F | ataggatccATGACAGCCATCATCAAAGAGATC |
| pCDNA3.1/PTEN-R | atactcgagTCAGACTTTTGTAATTTGTGTAT |

21bp targets against indicated genes

| Primer name 5′-3 | 5′-3′ |
| --- | --- |
| EZH2-F | GCTAGGTTAATTGGGACCAAA |
| EZH2-R | CCCAACATAGATGGACCAAAT |
| PTEN-F | CTAGAACTTATCAAACCCTTT |
| PTEN-R | CCACAAATGAAGGGATATAAA |
| Scramble | CCTAAGGTTAAGTCGCCCTCG |

NC: negative control.
